# A Study of the Crystallization, Melting, and Foaming Behaviors of Polylactic Acid in Compressed CO_2_

**DOI:** 10.3390/ijms10125381

**Published:** 2009-12-16

**Authors:** Wentao Zhai, Yoorim Ko, Wenli Zhu, Anson Wong, Chul B. Park

**Affiliations:** Microcellular Plastics Manufacturing Laboratory, Department of Mechanical and Industrial Engineering, University of Toronto, Toronto, Ontario, M5S 3G8, Canada; E-Mails: zhaiwt@mie.utoronto.ca (W.Z.); yoorim.ko@utoronto.ca (Y.K.); wlzhu@mie.utoronto.ca (W.Z.); awong@mie.utoronto.ca (A.W.)

**Keywords:** crystallization, polylactic acid, compressed CO_2_

## Abstract

The crystallization and melting behaviors of linear polylactic acid (PLA) treated by compressed CO_2_ was investigated. The isothermal crystallization test indicated that while PLA exhibited very low crystallization kinetics under atmospheric pressure, CO_2_ exposure significantly increased PLA’s crystallization rate; a high crystallinity of 16.5% was achieved after CO_2_ treatment for only 1 min at 100 °C and 6.89 MPa. One melting peak could be found in the DSC curve, and this exhibited a slight dependency on treatment times, temperatures, and pressures. PLA samples tended to foam during the gas release process, and a foaming window as a function of time and temperature was established. Based on the foaming window, crystallinity, and cell morphology, it was found that foaming clearly reduced the needed time for PLA’s crystallization equilibrium.

## 1. Introduction

For polymeric materials, especially for those with regular chain structures, crystallization is a general phenomenon and a very important process because it controls the polymer’s structural formation and thereby strongly influences the final products’ properties [1]. Polylactic acid (PLA) is one kind of biodegradable polymer being produced from annually renewable resources. There is a growing interest in it for many applications [2–6]. Unfortunately, the crystallization of linear PLA occurs too slowly for it to develop significant crystallinity. This is especially so during the nonisothermal conditions encountered in normal extrusion and injection molding processes, where it is hard to achieve high PLA crystallinity in a short time. PLA exhibits a glass transition temperature in the range of 50–60 °C. Below that temperature, PLA is rigid and brittle. Developing high crystallinity in PLA tends to increase the modulus and strength of elasticity as well as the service temperature [3–5]. Therefore, the question of how to enhance crystallization behavior and increase crystallinity has been widely discussed in the PLA processing field.

It has been reported that PLA samples with high crystallinity have been obtained by employing such methods as isothermal annealing [7–9], polymer blending [10–13] or compounding with inorganic particles [14–16], and strain-induced crystallization [17–19]. In particular, solvent-induced crystallization has produced PLA materials with high crystallization; however, that process uses organic solvents, which are difficult to eliminate from the final product and are not environmentally friendly. Hence, the final product would be inappropriate for use in medical applications. Technology based on supercritical CO_2_ is considered to be a solution to problems associated with the use of biomaterials involving polymers.

It is well established that CO_2_ is a readily available, inexpensive, and environmentally benign gas. It exhibits tunable liquid-like solubility and gas-like viscosity under supercritical fluid conditions, which can be readily accessed due to its relatively low critical point at *T*_c_ = 31.1 °C and *P*_c_ = 7.37 MPa. It has been shown that supercritical CO_2_ can swell and plasticize glassy polymers, leading to a depression of their glass-transition temperature (*T*_g_) to almost the same degree as is affected by solvents or vapors [20]. This remarkable depression in *T*_g_ means increased mobility in polymer chains, and hence allows for a fast relaxation, which subsequently resulting in a fast crystallization of the semicrystalline polymers, such as polyaryl ether ether ketone (PEEK) [21], PC [22–26], and even in the non-thermally crystallizable polymers, such as tert-butyl PEEK, at any temperature between *T*_g_ and the degradation temperature [27]. A wealth of papers dealing with CO_2_-induced crystallization in PLA [18,28,29] and PLLA [30–33] has been published in the past 10 years. However, very few of these have investigated the crystallization and melting behaviors of PLA at broad treatment temperature and gas pressure scopes.

Strain-induced crystallization is another factor that can enhance crystallinity development in polymers. According to previous report [17–19], crystal structure could be developed in amorphous PLA films when uniaxial or biaxial stretching was applied to the polymer. In polymer foaming processes with compressed CO_2_, cell nucleation occurs as a result of gas supersaturation, and then is followed by cell growth. A biaxial extensional flow is always formed in the cell walls during cell growth, which may initiate crystallization [18]. As a consequence, the foaming process may play a positive role in accelerating PLA crystallization. This phenomenon has been partially confirmed with PLLA by using batch foaming [30], where the crystallinity of PLLA is significantly increased from 5.2% (after CO_2_ saturation at 2 MPa) to 23.4% (after foaming at 100 °C for 30 s). However, it was hard to attribute all crystallinity development to the process of strain-induced crystallization, as exposure to a high temperature during foaming might also affect this behavior in PLLA. In another study, Mihai *et al*. [18] found that both CO_2_ exposure and the stretching process could induce PLA crystallization. However, it was difficult for them to separate the individual effect of CO_2_ exposure and the foaming process on the crystallinity of the final PLA samples.

In the present study, a linear PLA exhibiting a low crystallization rate at atmospheric pressure was selected. After being saturated with compressed CO_2_ at different pressures and temperatures for various lengths of time, the PLA’s crystallization and melting behavior were investigated to show the possible effects of processing parameters, such as treatment time, temperature, and pressure. The needed time for crystallization equilibrium and equilibrium crystallinity were determined, and the possible effects of temperatures on them were discussed. Also, PLA tended to foam after being saturated under some conditions, so a foaming window was established. Based on this window and the cell morphology of foams, we try to explain the individual effects of CO_2_-induced crystallization and strain-induced crystallization on the final crystallinity of PLA.

## 2. Results and Discussion

### 2.1. Isothermal Crystallization of PLA

**Figure 1.**
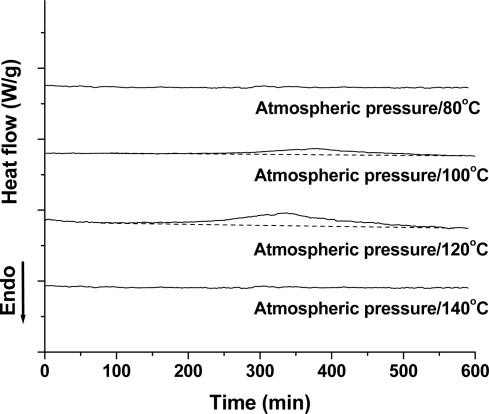
Isothermal crystallization of PLA at atmospheric pressure and various temperatures.

**Figure 2.**
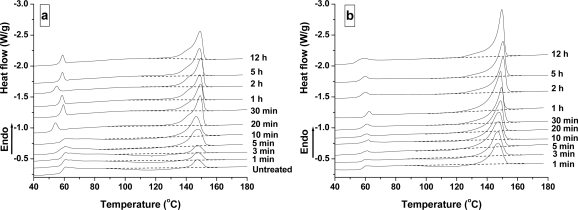
DSC curves of PLA samples treated under the CO_2_ pressure of 6.89 MPa at various times at 25 °C (a) and 100 °C (b).

PLA’s isothermal crystallization behavior was investigated using a regular DSC. Figure 1 shows the DSC curves of the isothermal crystallization of the PLA samples at different temperatures. At a low temperature of 80 °C, no obvious crystallization peak was found, indicating that the crystallization rate of PLA was very slow, and 10 h was not enough to induce its crystallization. As shown in the following figure (Figure 2), the *T*_g_ and *T*_m_ of the PLA is about 58 °C and 148 °C, respectively. Therefore, a temperature of 80 °C was too low to induce PLA crystallization within a short time due to low chain mobility. At 100 °C, however, PLA could crystallize, as observed by the small exothermal peak from 245 to 549 min. With further increasing the temperature to 120 °C, the crystallization rate became faster with crystallization occurring from 155 min, and the Δ*H*_c_ is bigger. When the temperature is increased to 140 °C, PLA crystallization becomes difficult, because the high chain mobility interferes with the regular arrangement of the polymer chain.

Based on these results, PLA crystallization was difficult. It was found that a longer time and narrower temperatures’ window might be needed to induce PLA crystallization. In addition, compared to 100 and 140 °C, it seems that 120 °C was a proper temperature to induce PLA crystallization under atmospheric pressure.

### 2.2. Cold-crystallization and Melting Behavior of PLA Treated by CO_2_

Amorphous PLA films were treated under compressed CO_2_, and the different processing parameters of temperature, pressure, and time were used during the treatment process. DSC was used to investigate the cold-crystallization and melting behavior of the PLA samples in this study.

#### 2.2.1. Effect of Time

Figure 2 shows the DSC curves of PLA samples treated at 25 °C and 100 °C at different times. As a reference, the untreated PLA sample is shown in Figure 2(a). It is known that the exothermic and endothermic peaks are attributed to the cold-crystallization and crystal melting of PLA during the DSC heating run, respectively. No crystallization occurred in the untreated sample, because the exothermic peak’s area around 98–136 °C was equivalent to the endothermic peak around 148 °C. After being treated by CO_2_ at 6.89 MPa and 25 °C for 1 min, the cold-crystallization area is present and very broad, and the area of exothermic peak is less than that of endothermic peak, indicating the presence of original crystal in CO_2_-treated PLA sample. This result demonstrated that PLA was induced crystallization after being treated by high pressure CO_2_ for 1 min. With treatment time increased from 1 to 10 min, the Δ*H*_c_ tends to decrease, while the Δ*H*_m_ tends to increase. At 20 min, the cold-crystallization disappears. When the time is more than 40 min, the shape of the melting peak does not change in an obvious manner, even if the treatment time goes up to 12 h. This indicates that the crystallization equilibrium of PLA under 6.89 MPa has been reached. At a high temperature of 100 °C, the CO_2_ treatment also significantly reduces the Δ*H*_c_, and no cold-crystallization is observed when the treatment time was longer than 20 min. In addition, the Δ*H*_m_ of PLA samples obtained at 100 °C is larger than at 25 °C, indicating that many more crystalline regions were formed when PLA samples were treated at a high temperature.

**Figure 3.**
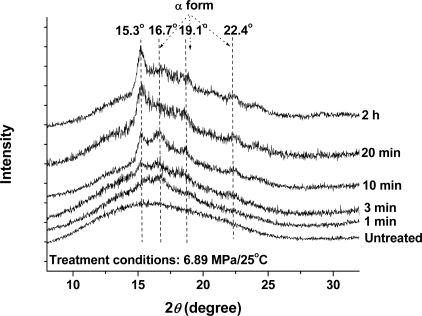
WAXD profiles of the untreated sample and the CO_2_-treated PLA samples at 6.89 MPa and 25 °C for 1–120 min.

Wide-angle X-ray diffraction (WAXD) measurements were taken to further confirm the crystallization behavior of PLA samples during the CO_2_ treatment, because cold-crystallization could be avoided during the WAXD test. Figure 3 shows the WAXD results of the PLA samples, where the same samples with the DSC measurements were used. The untreated PLA sample exhibits a broad diffraction peak at 2*θ* = 16.7°, which indicated that the crystallinity in this sample was too low to be detected by the WAXD measurement. This phenomenon was in agreement with the results measured by DSC. After the samples were treated by high-pressure CO_2_ at 25 °C for 1 min, a small crystalline peak appeared at 2*θ* = 16.7°, indicating the occurrence of CO_2_-induced crystallization. With increased treatment time, the peak intensity gradually increased. At a treatment time of 10 min, several new diffraction peaks at 2*θ* = 15.3, 19.1, and 22.4° appeared in the WAXD curve. As reported previously, the diffraction peaks at 2*θ* = 16.7, 19.1, and 22.4° belonged to α form [34]. Therefore, the diffraction peak at 2*θ* = 15.3° suggested the presence of a new crystal structure, suggesting a reorganization in the crystalline structure [18]. For CO_2_ treatment time longer than 20 min, the peak intensity did not change significantly, indicating that the CO_2_-induced crystallization had reached a plateau value. The WAXD result was consistent with the DSC, indicating that PLA was induced crystallization in compressed CO_2_ and the equilibrium crystallinity could be achieved as the treatment time had reached 40 min.

**Figure 4.**
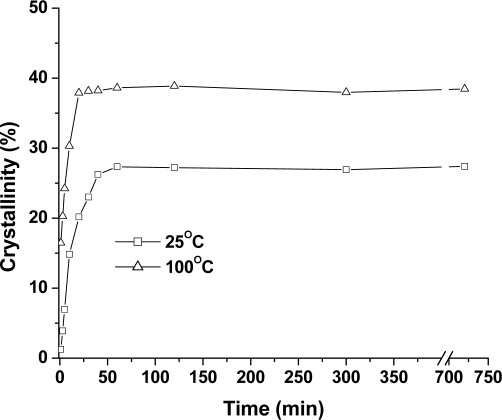
The crystallinity of PLA samples treated at 6.89 MPa and 25 °C and 100 °C.

Figure 4 summarizes the crystallinity results, obtained from DSC thermograms of the PLA samples treated in compressed CO_2_. At 25 °C, a small crystallinity of 1.3% is measured after treatment in compressed CO_2_ for only 1 min, and an equilibrium crystallinity of 27.4% is obtained after 40 min. In the meanwhile, a high crystallinity of 16.5% is obtained after 1 min of treatment at 100 °C. When the treatment time is further extended, the crystallinity increases quickly and reaches the maximum value at about 25 min. This demonstrated that the treatment temperature was a critical factor in crystallization behavior of the PLA samples. Also, it is noted that a higher crystallinity of 38.9%, was obtained at a higher temperature compared with 27.4% at a lower temperature.

It is known that supercritical CO_2_ can plasticize plastic, and decrease the *T*_g_ to several tenths of a degree. As a result, polymers can crystallize at a much lower temperature under supercritical CO_2_ than under atmospheric pressure. In this study, a temperature of 25 °C, which was much lower than the *T*_g_ of the original PLA (58 °C), enabled the PLA chain to form regular arrangements. This result demonstrated that CO_2_ treatment was effective in inducing PLA’s crystallization. In the past, polymers with poor crystallization ability, like polycarbonate (PC) [22–26] and polyaryl ether ether ketone (PEEK) [27] have been verified to crystallize in supercritical CO_2_. In the case of PC, however, a longer time (*i.e.*, about 2 h or even more), a high pressure, and a high temperature are normally needed to induce crystallization [25,26]. This finding suggests that, compared with PC, PLA possesses higher crystallization ability in compressed CO_2_.

#### 2.2.2. Effect of Temperature

**Figure 5.**
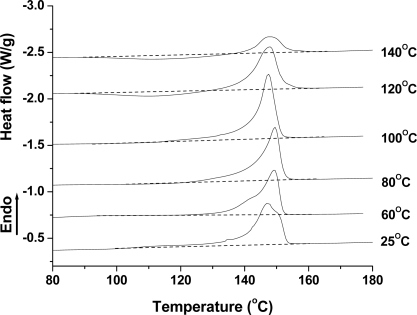
DSC curves of PLA samples treated at 6.89 MPa for 30 min.

Figure 5 shows the melting behavior of PLA samples treated at different temperatures for 30 min. At 25 °C, 60 °C, 80 °C, and 100 °C, no obvious cold-crystallizations are observed in the PLA samples. In addition, the endothermic area increases gradually as temperature increases. As the temperature rises to 120 °C and 140 °C, however, a broad cold-crystallization peak is presented in the DSC curves, and the endothermic area decreases correspondingly. It is noted that all PLA samples had one melting peak at 149 °C, and increasing temperatures further did not have an obvious effect on the melting point.

**Figure 6.**
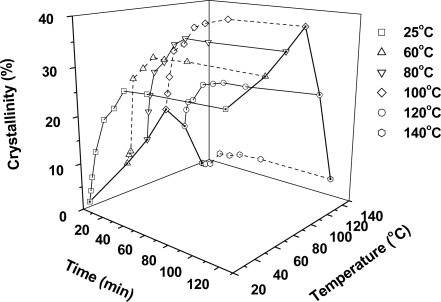
3D-map showing the crystallinity of PLA samples treated at 6.89 MPa at different temperatures at various times.

A 3D-map was used to summarize the crystallinity of PLA samples that were treated at 6.89 MPa and at different temperatures for different times, as shown in Figure 6. The data corresponding to 1 min and 120 min at different temperatures were connected respectively with a solid line to show the development of crystallinity with temperature and time. The data corresponding to longer times of 5 h and 12 h were not shown here. It is seen that the crystallinity of PLA increases quickly in the first 10 min, and then tends to level off as the time extends to 25–40 min. Temperature had an obvious effect on crystallization. At low temperatures, the crystallinity of PLA at both short and longer times clearly increased with increased temperature. For equilibrium crystallinity, the percentages and temperatures are 27.4% at 25 °C, 31.4% at 60 °C, 35.9% at 80 °C, and 38.9% at 100 °C. When the temperature exceeds 100 °C, however, the PLA’s crystallinity significantly decreases to 23.4% at 120 °C and 5.0% at 140 °C. Compared with equilibrium crystallinity, PLA crystallinity obtained at short times exhibited a much higher temperature dependency. For example, at 1 min, the crystallinity of PLA samples was 1.3% at 25 °C, 6.8% at 60 °C, 10.7% and 80 °C, 16.5% at 100 °C, 11.0% at 120 °C, and 0.0% at 140 °C. These results further indicate that a presence of maximum temperature for crystallization in PLA sample, which was similar to the crystallization behavior of other polyethers such as PC in supercritical CO_2_ [25,26].

Temperature is a critical parameter that affects the crystallization behavior of polymers in supercritical CO_2_, because an increased temperature leads to an increase in chain mobility that governs the polymer’s crystallization ability [22,26]. Due to the high plasticization effect of CO_2_, PLA could crystallize at a low temperature of 25 °C. With increased temperature, the crystallinity of PLA gradually increased. When the temperature reached the maximum crystallization point of 100 °C, the crystallinity of PLA tended to decrease significantly with temperature, because the chain mobility was too high that prevented the regular arrangement of the polymer chain. As mentioned above, the maximum crystallization temperature of PLA under atmospheric pressure was about 120 °C, and the introduction of high pressure CO_2_ shifted it down to 100 °C. The presence of a maximum crystallization temperature in supercritical CO_2_ has also been observed in other polyester such as PC [22,25]. According to another report, the maximum crystallization temperature of PC was about 140 °C [22], which was slightly lower than its *T*_g_, at 147 °C.

The melting peak of PLA exhibited no obvious change as the temperature was varied in the presence of compressed CO_2_, and the same result has been reported in PLLA by others [33]. However, this phenomenon was very different in the melting behavior of PC in supercritical CO_2_ [26], where the melting peak usually increased with the temperature. It is known that the increase in melting peak is associated with an increase in lamellar thickness [35]. Therefore, it is believed that the increased temperature did not facilitate the lamellar thickening process, which might be attributed to the special chain packing and crystallite arrangement within spherulite during PLLA crystallization in compressed CO_2_ [33]. In particular, the presence of a crystal morphological transition had been observed in PLA and PLLA during CO_2_ treatment by using WAXD [18] and light scattering measurements [33].

#### 2.2.3. Effect of Pressure

**Figure 7.**
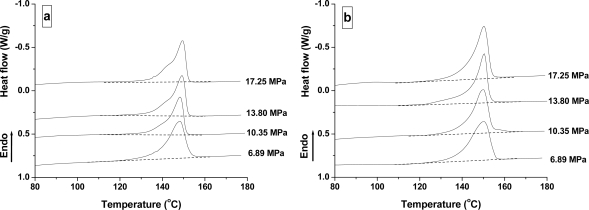
DSC curves of PLA samples treated under different CO_2_ pressures for 2 h at 25 °C (a) and 100 °C (b).

**Figure 8.**
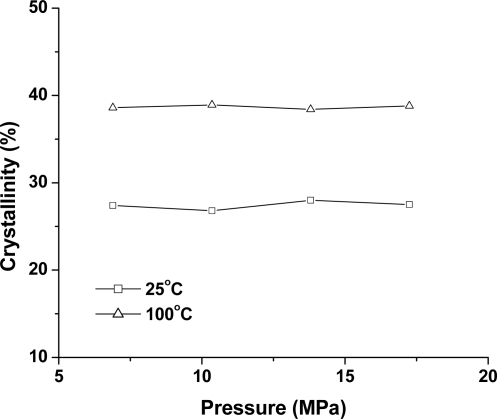
The crystallinity of PLA samples treated under different pressures for 2 h.

Figure 7 shows the DSC curves of PLA samples treated at different pressures and at 25 °C and 100 °C for 2 h. All PLA samples do not have a cold-crystallization peak at 6.89–17.25 MPa. Furthermore, with the increased pressure, the melting peak and endothermic area do not show any obvious change. This was further verified by the crystallinity as shown in Figure 8, where the crystallinity of PLA is 27.4% (6.89 MPa), 26.8% (10.35 MPa), 27.0% (13.80 MPa), and 27.5% (17.25 MPa) at 25 °C; and 38.9% (6.89 MPa), 38.3% (10.35 MPa), 38.8% (13.80 MPa), and 39.2% (17.25 MPa) at 100 °C. Therefore, there was almost no CO_2_ pressure dependence at the melting point and Δ*H*_m_, despite the presence of a transition in CO_2_ properties from a subcritical to a supercritical condition.

It is known that an increase in CO_2_ pressure tends to increase the mobility of polymer chains and lower *T*_g_ [32]. Therefore, transformation of the amorphous phase of polymer to a lower free energy crystalline structure is kinetically favorable [26]. However, increased CO_2_ pressure did not obviously affect the crystallization thermodynamics of PLA. A possible explanation for this may be that the compact crystallite structure restricted the solubility of CO_2_ in the crystal region, which then decreased the effect of CO_2_ pressure on crystalline thermodynamics [26]. A similar effect of CO_2_ pressure on the melting peak and crystallinity has been observed in PLLA [30,33] and PC [22,24–26]. It was found that the melting behavior of PLLA did not change at a CO_2_ pressure of 3–5 MPa [30] and 3–15 MPa [33]. At a much lower CO_2_ pressure of 1 MPa and 2 MPa, however, Wang *et al*. [30] reported that the crystallinity of PLLA was much lower than that of 3 MPa.

In summary, linear PLA exhibited a low crystallization rate, and 10 h were normally needed to induce PLA crystallization at atmospheric pressure. The CO_2_ treatment significantly increased the crystallization rate of PLA; a high crystallization of 16.5% could be obtained even within the short time of 1 min. It is believed that the high plasticization effects of compressed CO_2_ accelerated the crystallization rate of PLA.

### 2.3. The Induced Crystallization of PLA during the Foaming Process

During the gas releasing process, PLA samples could foam under some conditions due to the strong plasticization effects of CO_2_, even though a very thin CO_2_ film and a low depressurization rate was applied in these experiments. It is known that a biaxial extensional flow is usually formed in cell walls during cell growth, and this process facilitates strain-induced crystallization. Therefore, the final crystallinity obtained in foamed PLA samples tends to include both the contributions of CO_2_-induced crystallization and strain-induced crystallization. In this study, we tried to show their individual effects on the crystallization process.

**Figure 9.**
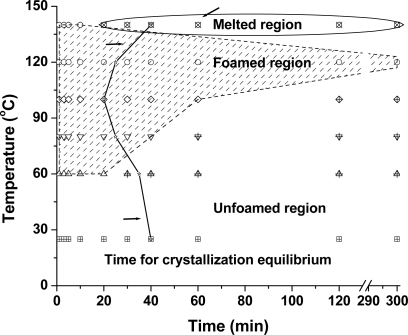
The foaming window of PLA samples with using a batch method, where the treatment pressure was 6.89 MPa. The average time for the crystallization equilibrium of PLA was 30 min.

Figure 9 shows the foaming window of PLA samples as a function of treatment temperature and time. The shadowed region in the figure shows where PLA could foam during the gas release process. Below the shadowed region, the PLA samples could not foam; while above the shadowed region, PLA tended to melt in a high-pressure vessel. It is seen that PLA could not foam at the low temperature of 25 °C. With an increase in temperature to 60 °C, however, foaming began to occur in PLA after being saturation within 20 min, and then could not foam again at the extended time. At a higher temperature, the foaming window of PLA tended to broaden gradually. For example, at 80 °C and 100 °C, foaming occurred after the PLA sample was saturated with compressed CO_2_ for 40 min and 1 h, respectively. At 120 °C, PLA could foam even after being saturated for 12 h. With temperature increased to 140 °C, however, PLA foamed only within 10 min, and then tended to melt completely when the time was more than 20 min. Under those conditions, no foams were obtained.

The curved line shown in Figure 9 represents the time required for crystallization equilibrium that was estimated by using the effects of time on crystallinity. The crystallization equilibrium time of PLA at 6.89 MPa exhibited an obvious temperature dependency. At 25 °C, 40 min is needed to reach crystallization equilibrium. With increased temperature, the required time linearly decreases to 35 min at 60 °C, 25 min at 80 °C, and 20 min at 100 °C. By further increasing the temperature, however, the required time increases to 25 min at 120 °C and about 40 min at 140 min, respectively.

As mentioned above, both CO_2_-induced crystallization and strain-induced crystallization contributed to crystallinity development in foamed PLA samples. For unfoamed conditions such as at 25 °C, however, it is believed that only CO_2_-induced crystallization contributed to the crystallinity of PLA because of the absence of foaming. Therefore, the data at 25 °C supplied a useful reference to describe the possible effect of polymeric foaming on the final crystallinity of PLA. In unfoamed conditions, a reduction of about 5 min in the time needed for crystallization equilibrium was observed by increasing the temperature of 35 °C (from 25 °C to 60 °C), resulting from the increased polymer chain mobility at high temperature. Once the foaming occurred, however, a reduction of about 15 min was realized by increasing the temperature of 20 °C (from 60 °C to 80 °C). These results demonstrated that the foaming also presented a positive effect in reducing the time requirement for crystallization equilibrium of PLA.

**Figure 10.**
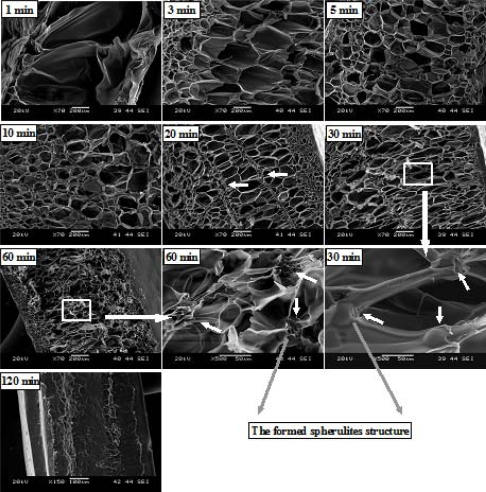
The cell morphology of PLA foams after saturation at 6.89 MPa at different times.

Figure 10 shows the SEM micrographs of PLA foams obtained after saturated at 6.89 MPa and 100 °C for different times. At the saturation time of 1 min, only a very few big cells are shown in the foam, which might be resulted from an insufficient CO_2_ exposure time; thus, CO_2_ solubility equilibrium was not reached. By increasing the time to 3 min, the cell size tended to decrease in the foam center, but a large amount of small cells were observed near the foam skin. At extended times of 5 min and 10 min, the cell size distributions become more uniform due to good gas dispersion in the PLA matrix. At 20 min, however, the obvious heterogeneous cell distribution is observed in the foam center, and lots of very small cells are present in those regions (shown in Figure 10 in the regions pointed by arrows). Similar cell morphology has been observed in PP [36] and polyester amide [37] foams with the foaming temperature that were slightly lower than their melting points, which was attributed to the occurrence of cell nucleation at/around crystal regions, *i.e.*, crystal phase induced heterogeneous cell nucleation. Based on cell morphology obtained after saturation of 10 min, as well as high crystallinity at 20 min, it is suggested that higher crystallinity might have been obtained at 20 min, before foaming. At the extended times of 30 min and 60 min, several spherical particles with a diameter of about 30–40 μm, which seems to be coated by polymer matrix, are observed in the junction of cells. Similar spherical particles have also been observed in the CO_2_ induced-crystallization’s polymeric foaming systems like PC [38], where spherical particles were thought of as spherulite crystal. Because no extra solid particles were added in the PLA foaming process, we believed that the same phenomenon occurred in the PLA foaming system. Furthermore, we believed that these spherulites should be formed during the CO_2_ saturation process. We made this claim based on two reasons: the first is that the cell wall stretching process during cell growth tended to make the cell wall and junction area thinner, while the particle size was much thicker than that of cell wall (30–40 *v.s.*, 5 μm); the second is that the stretching process that induced spherulite crystal formation was difficult. We also note that the thickness of foamed samples tended to decrease significantly from 30 to 60 min, and then to 120 min, where no foaming occurred.

**Figure 11.**
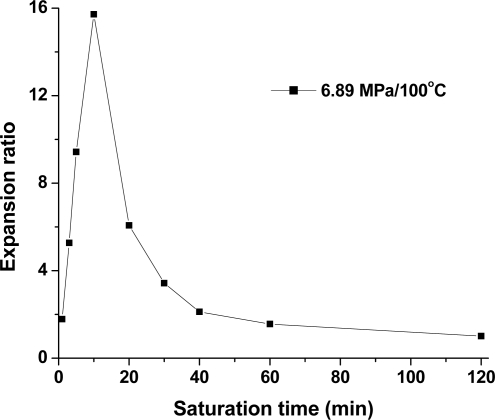
The expansion ratio of PLA foams after saturation at 6.89 MPa and 100 °C at different times.

Foaming-induced crystallization seems to be associated with foam expansion, and a high volume expansion ratio tended to induce high crystallinity in the PLA sample. Mihaela *et al*. [18] investigated the effect of biaxial stretching on the crystallinity of PLA. They found that a high stretch ratio of 9 induced high crystallinity of about 20% in the same PLA as was used in this study, while a low stretch ratio of 1–4 exhibited a small effect on the PLA’s crystallinity. These results suggest that the stretching process during cell growth might exhibit less effect in low expansion ratio foam compared with high expansion ratio foam. Figure 11 shows the expansion ratio of PLA foams as a function of treatment time. It is seen that the expansion ratio of PLA foams significantly increases within 10 min; that is, 3.21 at 1 min, 7.27 at 3 min, 11.43 at 5 min, and 15.72 at 10 min, and then quickly decreased to 6.07 at 20 min, 3.43 at 30 min, 2.12 at 40 min, 1.56 at 60 min, and to 1.01 at 120 s., respectively. The obvious foam expansion at 5 min and 10 min might contribute highly to crystallinity. This could be due to a rapid increase in the PLA foam’s crystallinity with the addition of the original crystallites formed during the CO_2_ saturation process. At the extended time of 30–60 min, PLA foams exhibited a low expansion ratio, because the high crystallinity that was formed during the CO_2_ saturation process, as confirmed by SEM micrographs, increased the stiffness of the polymer matrix and inhibited cell growth. Consequently, foam expansion might only slightly affect PLA’s final crystallinity. This result was consistent with the DSC measurement, where the equilibrium crystallization was reached at about 20 min, and no increase in crystallinity was observed by further extending the time up to 12 h. Therefore, the crystallinity formed by CO_2_ exposure determined the foaming behavior of PLA, and thus affected the contribution of strain-induced crystallization to the final crystallinity of PLA foams. To be specific, in the case of high CO_2_-induced crystallization, strain-induced crystallization had a small effect on the final crystallinity of PLA foams; while in low CO_2_-induced crystallization, strain-induced crystallization played an obvious role. Therefore, the PLA sample crystallized very quickly, and the short time required for equilibrium crystallization could be achieved after the sample was treated by the compressed CO_2_.

## 3. Experimental Section

### 3.1. Materials and Samples Preparation

A commercial linear PLA (Ingeo^™^, 2002D) in pellet form was provided by Natureworks LLC, USA. According to the supplier, its d-isomer content is 4.3%, melt flow rate is 5–7 g/10 min, and its density is 1.24 g/cm^3^. CO_2_ with a purity of 99.5% (Linde gas) was used as the physical blowing agent in all experiments. The PLA pellets were dried in the oven for 4 h at 80 °C before use. Then, polymer specimens with a thickness of about 0.3 mm were prepared by compression molding at 200 °C. The samples were cut into disc-shaped sheets for CO_2_ treatment.

### 3.2. High Pressure CO_2_ Treatment

A high-pressure apparatus was used for compressed CO_2_ treatment. The 0.3 mm-thick PLA film was placed in a high-pressure vessel preheated to the experimental temperature. The vessel was flushed with low-pressure CO_2_ for about 1 min and then pressurized to the desired value. Once the CO_2_ pressure reached the desired value, the timing began. At the end of the experiment, the vessel was released at a low depressurization rate, and the sample was removed quickly from the vessel.

### 3.3. Analysis

DSC measurements were carried out to determine the *T*_g_, cold crystallization temperature (*T*_c_) and melting peak (*T*_m_) by using a Q2000 (TA Instruments) that was calibrated with indium. All measurements were made at a heating rate of 10 °C/min over a temperature range of 20–200 °C in a dry nitrogen environment. The enthalpy of crystallization on heating Δ*H*_c_ and melting enthalpy Δ*H*_m_ were measured. The crystallinity of PLA CO_2_-treated samples was calculated by [(Δ*H*_m_ Δ*H*_c_)/Δ*H*_f_] × 100%, where Δ*H*_f_ is the theoretical heat of fusion of 100% crystalline PLA with a value of 93 J/g [39]. Isothermal crystallization from the amorphous state was used to measure the crystallization rate of PLA. The samples were first heated to 200 °C at a rate of 10 °C/min to eliminate any thermal history, and then quickly cooled to the present crystallization temperature to initiate isothermal crystallization. In the isothermal crystallization study, the samples were held at the crystallization temperature for 10 h. For CO_2_-treated PLA samples, the first heat scanning curve was used to calculate crystallinity.

WAXD measurements were taken on a Siemens D5000 diffractometer equipped with a Kevex solid-state detector that uses a Cu Kα radiation source with a wavelength of 1.54Å. The measurements were performed at 50 kV and 35 Ma. The data were recorded in reflection mode at a range of 2*θ* = 5–35° using a STEP scan mode. The step size was 0.02 degrees and the counting time was 2.0 s per step.

After being saturated by compressed CO_2_, PLA samples tended to foam under some conditions during the gas release process. Cell morphologies of the foamed samples were observed with a JEOL JMS 6060 scanning electron microscope (SEM). Prior to the SEM observation, the samples were freeze-fractured in liquid nitrogen and sputter-coated with platinum. The volume expansion ratio of the polymer foams can be calculated according to Equation (1):

(1) $$\varphi \frac{\rho }{\rho_{f}}$$

where *ρ* and *ρ*_f_ are the mass densities of the samples before and after foaming, respectively, which were measured by the water displacement method in accordance with ASTM D792.

## 4. Conclusions

Due to the low chain mobility, linear PLA exhibits a very low crystallization rate under atmospheric pressure. This was confirmed by the present study, where about 10 h were needed to isothermally induce PLA crystallization at a suitable temperature of 120 °C. The crystallization and melting behavior of PLA in high pressure or supercritical CO_2_ has been investigated by changing the treatment time, temperature, and gas pressure. It was found that a high crystallinity of 16.5–27.4% could be developed after CO_2_ treatment at 100 °C for only 1 min at a low temperature of 25 °C. Moreover, the time for crystallization equilibrium significantly shortened to 20–40 min. These results indicated that the high plasticization effect of compressed CO_2_ effectively increased the mobility of the polymer chain, and thus accelerated the crystallization rate and broadened the crystallization window of PLA. An optimum crystallization temperature of 100 °C was observed during the PLA crystallization at 6.89 MPa. At a lower temperature, a temperature increase tended to increase the crystallinity of PLA, while at a higher temperature, a reversed trend was observed. DSC curves showed that cold-crystallization tended to decrease and then disappear with increased crystallinity. Only one melting peak could be found in the DSC curve, which was slightly depended on treatment times, temperatures, and pressures. These results indicated that the changed treatment conditions did not affect the perfection of crystals, and thus didn’t affect the lamellar thickening process of crystal domain during the crystallization of PLA. PLA samples tended to foam during the gas release process under some conditions, and a foaming window as a function of temperature and timing was established in this study. Based on the crystallinity, foaming window, and cell morphology, it was found that the occurrence of foaming reduced the time for the crystallization equilibrium of PLA. For the PLA samples with high crystallinity obtained from CO_2_ exposure, however, the increased matrix modulus suppressed foam expansion. Consequently, the strain-induced crystallization that resulted from the PLA foaming process only had a slight effect on the final crystallinity of the PLA samples.
